# Practical Notes and Formulæ

**Published:** 1882-09-20

**Authors:** 


### PRACTICAL NOTES AND FORMULÆ.

**Excoriated Papular Eczema.**—It is a case where strong remedies are needed. There are two or three lotions that may be used. Carbolic acid and thymol both act happily in such cases. The alkaline tar lotion also might be used, according to the following formula—

℞ Picis liquidæ.............................. ʒ ij,

Potassæ.............................. ʒ j,

Aquæ.............................. f. ʒ v.

M. Sig. Dilute a teaspoonful into two or four ounces of water, and use freely as a lotion.

In this case, however, we will first employ the carbolic acid, prepared as follows—

℞ Acidi carbolici.............................. f. ʒ ij,

Glycerinæ.............................. f. ℥ ss,

Alcoholis.............................. f. ℥ ij,

Aquæ.............................. ad O j.

M. This will be applied freely, three times a day, for ten minutes at a time. The application may cause smarting, but its use should be persisted in for three or four days. At the end of this time the more acute symptoms will have subsided, and we will substitute the lotion by an ointment of white precipitate or calomel, thirty grains to the ounce of cosmoline and oxide of zinc ointment. The use of the lotion may, perhaps, be repeated from time to time. It will, probably, require several months to effect a complete cure.—*Med. and Surg. Rep.*

**Camphorated Chloro-Tannate of Iodine.**—The above named preparation to be used as a topical application to bleeding ulcers and cancers of the cervix uteri, is made as follows—

℞ Chloral hydrate.............................. ʒ i,

Iodine.............................. ʒ ss,

Oil of camphor.............................. ʒ vi.

M. S. ft. sol., et. adde.

Tannic acid q. s. to bring the mixture to the consistence of thick syrup.

For hemorrhagic ulcers and cancers of the cervix uteri, we have found the above preparation an excellent application, both as a hemostatic, deodorizer and alterative. For bleeding cancers, we use the medicine pure, by charging a pledget of candlewick with the mixture, and placing it against the affected part, filling in the vagina below with dry clean wicking. The application should be renewed every day, as long as hemorrhage threatens or bad odor persists; and before each dressing, the parts should receive a prolonged hot syringe-bath, with a dilute solution of chloride of zinc. Then more emollient applications—as the comp. iodoform ointment—will be in order.

A good formula for

COMP. IODOFORM OINTMENT.

| | | |
|---|---|---|
| ℞ | Iodoform, | aa ʒ i, |
| | Ergotin | |
| | Pine tar | |
| | Balsam, | |
| | Vaseline | ℥ i. |

M. S., ft. ung.

In the treatment of ulcers, the first named preparation may be mitigated with vaseline to suit the case. Of course, each case should receive appropriate constitutional treatment, both medical and hygienic.—Dr. Q. C. Smith in *Southern Practitioner*.

**Cracked Nipples.**—Le Paris Medical publishes a number of formulæ, which are recommended in this complaint—

| | | | | |
|---|---|---|---|---|
| No. 1. | ℞ | Cosmoline | ʒ xiiss, | |
| | | Liquid balshm Peru | ʒ i¼. | M. |
| No. 2. | ℞ | Oxide of zinc | ʒ i¼, | |
| | | Cold cream of cosmoline | ʒ x. | M. |
| No. 3. | ℞ | Glycerole of starch | ʒ viiss, | |
| | | Oil of cade | *m* xlv. | M. |
| No. 4. | ℞ | Cacoa butter | ʒ iiss, | |
| | | Oil sweet almonds | ʒ ss, | |
| | | Extract of rhatany | *m* xv. | M. |
| No. 5. | ℞ | Gutta percha | ʒ j, | |
| | | Pure chloroform q. s. to dissolve. | | |

By anointing the excoriations with this a slight film is formed, which will not become detached, even after sucking.—*Med. and Surg. Rep.*

**Diphtheria.**—

| | | |
|---|---|---|
| ℞ | Tinct. ferri chlor | ʒ iv, |
| | Quinia sulph | grs. xxxii, |
| | Elix. simp | ℥ i, |
| | Aquæ add | ℥ viii. |

Sig. Tablespoonful every four hours in water.

| | | |
|---|---|---|
| ℞ | Tinct. ferri chlor | ℥ i, |
| | Chlor. potassa | ℥ i-ss, |
| | Acidi carbol | gtt. xl, |
| | Glycerine | ℥ i, |
| | Aquæ add | ℥ viii. |

Sig. Swab the throat every hour; use a probang.—D. W. Ham, M. D., in *Medical Brief*.

**Constipation.**

R Leptandrin ........................................ grs. xx,
Ext. colcoynth comp .............................. grs. xx,
Powd. podophyllin ................................. grs. x,
Syr. rhei aromat ................................... ℥ iv.

Sig. Teaspoonful three times a day.—*Ex.*

**Fuming Inhalations in Asthma.**—

R Potas. nitras ........................................ ʒ ss,

Pulv. anisi fruct ....................................... ℥ ss,

Pulv. stramonii fol ..................................... ℥ i.

M. A thimbleful placed on a plate is pinched into a conical shape and lighted at the top—burns like a pastile and held near the patient who inhales the fumes.—*Phila. Med. Times.*

**Blackberry Extract in Diarrhœa.**—Dr. B. F. Humphreys, (in Medical Brief) recommends the following in diarrhœa and dysentery:

R Ext. rubi fluid ........................................ ʒ iiij.

Syrup, rhei aromat ..................................... ℥ j,

Ext. hamamelis fluid .................................... ʒ iij,

Tinct. opii ................................................ ʒ ij.

M. A teaspoonful every two, three or four hours. A child should be given five drops for every year of its age. Blackberry is an old and popular remedy in intestinal disorders. The above is an agreeable method of administering it.—*Ex.*

**A New Remedy—Jamaica Dogwood.**—Mrs. H., æt. 60, had suffered with asthma for forty years, almost daily, a chronic case aggravated by a change of temperature. I saw her November 10, 1880; countenance pallid, respiration labored, and was propped up in bed by pillows and chairs. She said, "Doctor, can you help me?" I answered, "I will try." "You think differently, Doctor, from Dr. R.," she said, "for he says I can't live long." I prescribed—

R Jamaica dogwood, fl. ext ............................ ℥ ij,

Syr. Ipecac ................................................ ʒ j,

Syr. aurant. cor ........................................... ℥ ss.

M. Sig. Teaspoonful every hour until the paroxysm shall cease; afterwards, once in four hours until morning.

The medicine gave almost instant relief. Next morning I continued the same prescription, alternating with a teaspoonful of the tincture of cinchona every six hours. I visited the patient twice subsequently and discharged the case. The lady has removed to a distant State, but she always keeps the medicine on hand, and she informs me in a recent letter that it always cuts the paroxysm short.—*Brief—Therapeutic Gaz.*

